# Cardiomyocyte-Specific miRNA-30c Over-Expression Causes Dilated Cardiomyopathy

**DOI:** 10.1371/journal.pone.0096290

**Published:** 2014-05-02

**Authors:** Wino J. Wijnen, Ingeborg van der Made, Stephanie van den Oever, Monika Hiller, Bouke A. de Boer, Daisy I. Picavet, Iliana A. Chatzispyrou, Riekelt H. Houtkooper, Anke J. Tijsen, Jaco Hagoort, Henk van Veen, Vincent Everts, Jan M. Ruijter, Yigal M. Pinto, Esther E. Creemers

**Affiliations:** 1 Heart Failure Research Center, Academic Medical Center, Amsterdam, The Netherlands; 2 Interuniversitair Cardiologisch Instituut Nederland (ICIN-NHI), Utrecht, The Netherlands; 3 Department of Cell Biology and Histology, Leeuwenhoek Center of Advanced Microscopy (LCAM), Academic Medical Center, Amsterdam, The Netherlands; 4 Laboratory Genetic Metabolic Diseases, Academic Medical Center, Amsterdam, The Netherlands; Maastricht University Faculty of Health, Medicine, and Life Sciences, Netherlands

## Abstract

MicroRNAs (miRNAs) regulate many aspects of cellular function and their deregulation has been implicated in heart disease. MiRNA-30c is differentially expressed in the heart during the progression towards heart failure and *in vitro* studies hint to its importance in cellular physiology. As little is known about the *in vivo* function of miRNA-30c in the heart, we generated transgenic mice that specifically overexpress miRNA-30c in cardiomyocytes. We show that these mice display no abnormalities until about 6 weeks of age, but subsequently develop a severely dilated cardiomyopathy. Gene expression analysis of the miRNA-30c transgenic hearts before onset of the phenotype indicated disturbed mitochondrial function. This was further evident by the downregulation of mitochondrial oxidative phosphorylation (OXPHOS) complexes III and IV at the protein level. Taken together these data indicate impaired mitochondrial function due to OXPHOS protein depletion as a potential cause for the observed dilated cardiomyopathic phenotype in miRNA-30c transgenic mice. We thus establish an *in vivo* role for miRNA-30c in cardiac physiology, particularly in mitochondrial function.

## Introduction

Since the initial discovery, it has become evident that miRNAs have a profound impact on cellular function [1]–[4]. MiRNAs function by decreasing transcript stability or inhibiting translation of their target mRNAs into protein through hybridisation with complementary sequences [5]. This results in decreased protein expression of the miRNA target, which makes miRNAs important regulators of protein synthesis, placing them at a central position in the maintenance of cellular and tissue homeostasis.

Several miRNAs have already been implicated in cardiac disease. For example, miRNA-29 regulates the expression of pro-fibrotic genes and is differentially expressed after myocardial infarction in mice [6], while experimentally overexpressed miRNA-133a protects against pressure overload-induced cardiac remodelling [7]. MiRNA-34a has been identified to control cardiac ageing via its direct targeting of a regulator of protein phosphatase 1 (i.e. PNUTS), thereby inducing cardiomyocyte apoptosis and telomere shortening [8]. Regarding mitochondrial function, the miRNA-199a-214 cluster was found to impair mitochondrial fatty acid oxidation via downregulation of PPARδ [9]. MiRNA-106b induces mitochondrial dysfunction in C2C12 myotubes through targeting of mitofusin-2 [10]. Interestingly, nuclear encoded miRNA-181c has been shown to decrease the mitochondrial encoded mt-COX1 gene expression [11], illustrating the intricate interactions of miRNAs and the genome.

MiRNA-30c belongs to the miRNA-30 family, which consists of five members that are ubiquitously expressed, all of which are among the most highly expressed miRNAs in the heart. Since the seed region is identical between members of the miRNA-30 family, it can be expected that there is a substantial overlap in the targets that they regulate. As a consequence, functional redundancy is expected between the miRNA-30 family members. In cultured cells, miRNA-30c is found in cardiomyocytes as well as in fibroblasts [12]. *In vitro*, miRNA-30c has been implicated in the regulation of distinct cellular processes, ranging from cardiac fibrosis and cardiomyocyte hypertrophy to mitochondrial function, apoptosis, and cell proliferation. Therefore, deregulation of this miRNA is expected to have profound effects on cardiac function. However, little is known on the role of the miRNA-30 family in the heart *in vivo*. Uncovering the exact role of miR30 *in vivo* is highly relevant as miRNA-30c was identified as the top candidate for inducing cardiomyocyte hypertrophy in an unbiased miRNA mimic screen in neonatal rat cardiomyocytes [13]. In addition, *in vitro* studies from our laboratory have implicated miRNA-30c as a regulator of cardiac fibrosis by its direct targeting of connective tissue growth factor (CTGF) [12], a finding that until now has not been verified *in vivo* [14]. Another direct *in vitro* target of miRNA-30c that regulates cardiac fibrosis is the plasminogen-activator-inhibitor-1 (PAI-1), a serine protease inhibitor that prevents the activation of matrix metalloproteases [15], [16]. Members of the miRNA-30 family also affect mitochondrial fission and apoptosis in cultured neonatal cardiomyocytes, an effect attributed to miRNA-30c targeting of p53 [17]. Assays with cancer cell lines show the inhibitory action of miRNA-30c on cell proliferation, possibly mediated via direct targeting of metastasis-associated gene-1 (MTA1) [18]. In addition, in zebrafish, miRNA-30 overexpression with mimic sequences leads to excessive blood vessel sprouting, showing the ability of this miRNA to induce angiogenesis *in vivo*. This angiogenic effect is mediated by DLL4, a membrane-bound ligand that inhibits angiogenesis via an effect on Notch-signalling [19]. Furthermore, the finding that miRNA-30c influences the effects of angiotensin-II implies its involvement in regulation of cardiac function during states of chronic pressure overload [20]. Finally, miRNA-30c has been implicated in the regulation of lipid metabolism via its effects on microsomal triglyceride transfer protein (MTP) in the liver. In this regard, elevated miRNA-30c levels were found to have therapeutic potential for the treatment of hyperlipidemia [21].

Several studies revealed a downregulation of miRNA-30c in the mouse heart during pressure overload-induced heart failure [12], [22]. In human heart disease, where the underlying aetiology is more heterogeneous than in experimental models of heart failure, the deregulation of miRNA-30c is less consistent. Whereas some studies show decreased expression of miRNA-30c in dilated cardiomyopathy (DCM), others have found trends towards an upregulation in this disease state [12], [23], [24]. Taken together, there are many studies that implicate miRNA-30c in cardiac disease: it is regulated in disease *in vivo* and several important functions have been ascribed to it *in vitro*. However, its *in vivo* effects on the heart have not yet been clearly established.

Therefore, we set out to further investigate the role of miRNA-30c in cardiac physiology. Here, we describe the results of our *in vivo* studies in miRNA-30c transgenic mice. We show that cardiomyocyte-specific miRNA-30c overexpression results in a dilated cardiomyopathy. Before the onset of the phenotype, we found signs of mitochondrial dysfunction, with a depletion of oxidative phosphorylation complex proteins. In conclusion, our study shows that elevated miRNA-30c expression interferes with normal cardiac function, at least partially via its involvement in regulating mitochondrial function in cardiomyocytes *in vivo*.

## Materials and Methods

### Animal experiments and generation of miRNA-30c transgenic mice

All animal studies were approved by the Institutional Animal Care and Use Committee of the University of Amsterdam (approval no DCA101423) and in accordance with the guidelines of this institution and the Directive 2010/63/EU of the European Parliament, and carried out in compliance with the Dutch government guidelines. For transgenesis, the murine pre-miRNA-30c-1 from chromosome 4 (GenBank acc. NT_039264.6; bases 20893658-20893266) was cloned into the αMHC expression plasmid (pBSII-SK+ containing the murine αMHC promoter region; GenBank acc. U71441) with SalI. The plasmid backbone was removed by digestion with SstII, generating a ∼6.5 kb fragment containing the αMHC promoter region, pre-mmu-miRNA-30c-1 genomic insert and the human growth hormone polyA sequence. Transgenic FVB mice were generated by pro-nuclear oocyte injection. Offspring was tested for miRNA-30c expression and 2 founders were crossed back into a C57Bl/6JOlaHSD background. Data were obtained from line B unless mentioned otherwise. For sacrifice, mice were anaesthetized with a CO2/O2 mixture and subsequently killed by cervical dislocation.

### *In situ* hybridization

For *in situ* hybridization staining we used 7 µm thick paraffin sections. These were dewaxed, rehydrated and 5′ equilibrated in phosphate-buffered saline (PBS). Sections were 15′ incubated with 1-Methylimidazole buffer (1% v/v 1-Methylimidazol pH 8.0, 0.3 M NaCl), 30′ incubated in EDC (0.16 M 1-ethyl-3-(3-dimethylaminopropyl carbodiimide) in 1-Methylimidazole buffer), washed in PBS, proteinase-K treated for 5′ at 37°C, washed with PBS, 15′ incubated in 1-Methylimidazole buffer, 30′ incubated in EDC, washed in PBS, 10′ fixed in 4% parafix, washed in PBS, 20′ incubated in 3% H_2_O_2_ in PBS, and washed in PBS. Then sections were prehybridized for 30′ in hybridization mix (10 mM Hepes pH 7.5, 600 mM NaCl, 50% v/v Formamide, 1 mM EDTA, 0.1% w/v Ficoll 400, 0.1% polyvinylpyrolidon, 0.1% BSA, 500 ug/ml Haring sperm DNA) at 60°C, 1.5 hr hybridized in 5′ pre-boiled hybridization mix with 500 nM LNA probes (Ribotask; sequences are shown in Table S3) at 60°C and then washed at 60°C in 2x SSC (0.3 M NaCl, 30 mM Trisodium Citrate), 0.5x SSC and 0.2x SSC successively. Detection of the bound probes was performed by 30′ blocking with 2% w/v blocking powder (Roche) in PBS-T, 45′ incubation with anti-FITC-HRP (Roche) 1∶4 in blocking solution, washing with PBS-T, 5′ Tyramide Signal amplification (TSA, PerkinElmer, NEL741), washing in PBS-T, 45′ incubation with anti-FITC-AP 1∶200 in blocking solution, washing in PBS-T, washing in NTM solution (100 mM Tris pH 9.5, 100 mM NaCl, 50 mM MgCl_2_), incubation in NBT/BCIP (Roche) 1∶50 in NTM solution, washing in bi-distilled water, 15′ incubation in nuclear red solution, washing in distilled water followed by dehydration of the slides and mounting with Entellan. Images were acquired with a 20X objective with a light microscope (Zeiss Axiophot).

### Northern Blot

Probes for miRNA-30c and U6 (loading control) were labelled using the miRNA StarFire Kit (Integrated DNA Technologies) and purified on Illustra ProbeQuant G50 Micro-columns (GE Healthcare) according to the manufacturer's protocol. 3 µg total RNA (TRIzol isolated) was separated on a 16% acrylamide gel, transferred to Hybond N+ membranes and UV-crosslinked (0.200 J/cm^2^). Labeled probes were hybridized for 30′ at 39°C in hybridization buffer (7% SDS, 200 mM Na_2_HPO_4_, pH 7.2), subsequently washed at 42°C with 2x SSPE buffer (0.36 M NaCl, 200 mM NaH2PO4, 20 mM EDTA, 0.1% DEPC, 0.1% SDS, pH 7.4) and exposed to Phospho-image film overnight.

### Echocardiography

Left ventricular function and dimensions were determined by transthoracic 2D echocardiography using a Vevo 770 Ultrasound (Visual Sonics) equipped with a 30-MHz linear array transducer. Mice were sedated on 4% isoflurane and anesthesia was maintained by a mixture of O_2_ and 2.5% isoflurane. M-mode tracings in parasternal short axis view at the height of the papillary muscle were used to measure LV internal diameter at end-systole and end-diastole. Fractional shortening was calculated from these internal diameters using the following equation: ((LV end-diastolic diameter - LV end-systolic diameter)/LV end-diastolic diameter) ×100%.

### Quantitative Real Time PCR (qPCR)

For qPCR, RNA was isolated from tissue or cells using TRIzol (Invitrogen 15596–026) according to the manufacturer's protocol. Subsequently, 200 ng RNA was treated with DNAse I (Invitrogen 18068–015). cDNA was synthesised using Superscript II reverse transcriptase (Invitrogen 18064–071) and diluted 4 times with milliQ water. Quantitative real time PCR was performed on a Lightcycler 480 (Roche) using SYBR green (Roche 04887352001). For miRNA quantification cDNA was synthesized with the miScript reverse transcription kit (Qiagen 218061), diluted 8 times with milliQ and qPCR was performed with High Resolution Melting Master (Roche) on a Lightcycler 480 according to the manufacturers' protocols. To validate miRNA-30c expression levels we also performed qPCR with the Taqman primer assay according to the manufacturers' protocol. Primer sequences are listed in Table S3. Analysis of qPCR data was performed using LinRegPCR analysis software [25], [26].

### Histology and fibrosis analysis

Hearts were dissected and immediately fixated overnight in 4% paraformaldehyde solution, washed with 70% ethanol, dehydrated and embedded in paraffin. Tissue sections were cut at 7 µM and deparaffinized prior to staining using xylene and a decreasing alcohol series. Sections were stained with Mayer's Haematoxylin for 8′, followed by flushing with tap water for 8′; Azophloxin stain was incubated for 3′, followed by washing for 1′ with water.

For PicroSirius Red staining, sections are incubated with 0.1% Sirius Red in picric acid for 60′, followed by 2′ in 0.01 N HCl. Dehydration was performed in an increasing alcohol- xylene series and slides were mounted with Entellan (Merck). Images were acquired with a Leica DM6000 microscope. Interstitial fibrosis quantification was performed using a customized macro in ImagePro Plus 6.20. After randomization of the images, epicardial and perivascular fibrosis was excluded from analysis. Fibrotic area is expressed as percentage of total tissue area and based on the pooled area of 20 randomized images per heart.

### Cell counting and volume analysis

Cardiac tissue sections were immunohistochemically stained for cardiac troponin I (Chemicon MAB1691) and Nkx2.5 (Santa Cruz sc-14033). Nuclear staining was performed with DAPI. Images were acquired with a Leica DM6000 microscope and stitched to create full cardiac cross-sections. A manual threshold was applied to determine the cTnI positive area. Within this area Nkx2.5 positive nuclei were automatically detected [27]. Total cardiac cross-sectional area was calculated based on the cTnI positive area and mean cardiomyocyte volume was derived from the total cardiac area and the number of Nkx2.5-positive nuclei. Data are based on analysis of 7 wildtype and 7 miRNA-30c transgenic hearts.

### Microarray and pathway analysis

Microarrays were performed by ServiceXS (Leiden, The Netherlands) on an Affymetrix Genetitan mouse 430 Plus 2.0 platform according to the manufacturer's protocol. RNA from wildtype mice and transgenic littermates of 4 (N = 3) and 20 (N = 2) weeks old was isolated from left ventricular tissue using TRIzol (Invitrogen 15596–026) and treated with DNAse I (Invitrogen 18068–015) according to the manufacturer's protocol. RNA quality was tested with the bioanalyzer (Agilent) and all samples had a RNA integrity number of 7 or higher. The expression data from the arrays was analyzed using the affylm graphic user interface for Bioconductor in R. Pathway analysis on significantly down- and upregulated genes (p-value < 0.05) was performed with the web-based DAVID functional annotation tool version 6.7 [28], [29].

### Protein analysis

Protein was extracted from LV tissue in RIPA buffer (50 mM Tris-HCl pH 8.0, 150 mM NaCl, 1% Igepal CA-630, 0.5% Sodium deoxycholate, 0.1% sodium dodecyl sulfate) with addition of protease inhibitor cocktail (Roche) and 1 mM Sodium Orthovanadate. Protein concentrations were measured using the BCA protein assay kit (Pierce) according to manufacturer's protocol.

For western blots, protein was diluted in a sample buffer (17.5% glycerol, 6% SDS, 250 mM Tris-HCl pH 6.7, 10% β-mercapto-ethanol) and denaturated 5′ at 95°C (GAPDH) or 50°C (OXPHOS). 60 µg of protein was loaded and separated on a 15% acrylamide gel and transferred to a methanol activated polyvinylidene fluoride (PVDF) membrane (Millipore). This membrane was blocked 1 hr in 5% protifar in TBST and incubated with the first antibodies overnight at 4°C (OXPHOS cocktail 1∶1000 (Abcam ab110413); GAPDH 1∶5000 (Bioconnect RDI-TRK5G4-6C5)). The following day membranes were washed in TBST and at least 2 hr incubated in the secondary antibody at RT (1∶5000, HRP-linked). Bands were detected using the ECL prime western blotting detection reagent (Amersham) and images acquired using the ImageQuant LAS4000 (GE Healthcare).

### Electron microscopy

Cardiac tissue was isolated and immediately prefixed in 1% glutaraldehyde, 4% paraformaldehyde in 0.1 M sodium cacodylate. Left ventricular samples were excised and postfixed with a solution of 1% OsO_4_ in cacodylate buffer. Subsequently the specimens were dehydrated in an alcohol series and embedded into Epon. Ultrathin sections were collected on formvar-coated grids and counterstained with uranil acetate and lead citrate. Images were acquired at a magnification of 6000x for overviews and 20500x for further analysis with a FEI technai 12 transmission electron microscope.

Double-blinded image analysis was performed with QWIN using an algorithm for manual mitochondrial annotation. Cross-sectional mitochondrial area was determined by annotation of the double-layered mitochondrial boundaries. Image border-touching objects were excluded from analysis. Average individual profile areas were obtained by dividing the total mitochondrial area by the number of individual double-layered objects. Quantitative data are based on the analysis of 18 randomized images per heart, 3 hearts per group.

### Mitochondrial abundance and citrate synthase activity

Mitochondrial DNA (mtDNA) abundance was quantified as previously described [30]. In short, total DNA was extracted from mouse heart tissue lysates, using the QIAamp DSP DNA Mini Kit (Qiagen). Quantitative PCR was performed with mitochondrial DNA- and nuclear DNA-specific primers for COX2 and UCP2 respectively (Table S3), on a Roche Lightcycler 480 using Roche SYBR-green mastermix.

Citrate synthase activity was measured in mouse heart tissue lysates as previously described [31].

### Statistics

All statistical testing was performed using unpaired two-sided Student's T-test unless mentioned otherwise. Effects on LVID,d and LVID,s were analysed by 2-way ANOVA for repeated measures. P-values ≤0.05 were considered statistically significant. Error bars represent standard error of the mean (s.e.m.).

## Results

### MiRNA-30c is expressed in cardiomyocytes and non-myocytes

**Figure 1:**
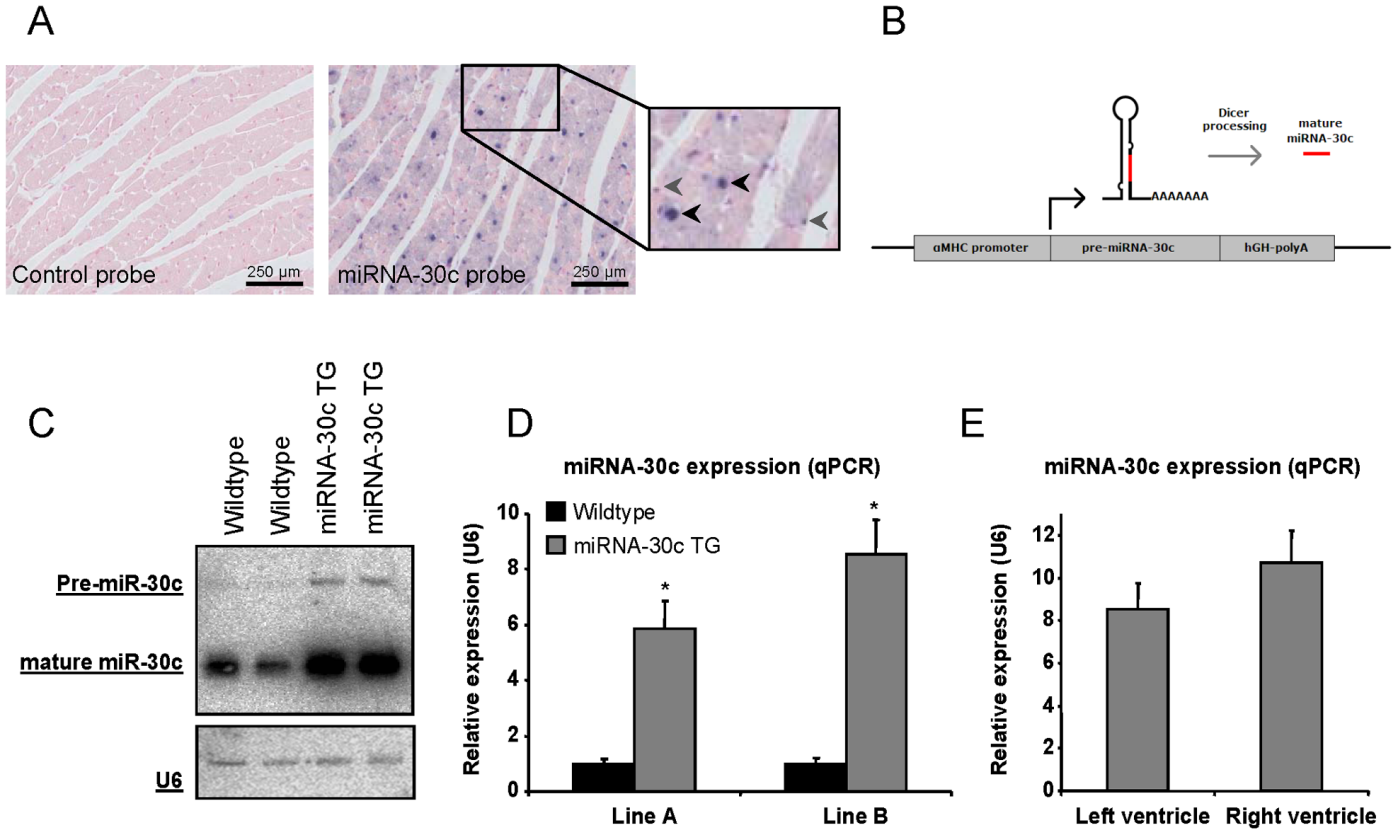
MiRNA-30c expression in the heart and the generation of αMHC-miRNA-30c transgenic mice (miRNA-30c TG). (a) MiRNA-30c in situ hybridization on adult wildtype hearts shows expression in the nuclei of both cardiomyocytes (black arrowheads) and interstitial cells (grey arrowheads). The cytoplasm is also miRNA-30c positive. (b) Schematic overview of the miRNA-30c overexpression construct used for the generation of transgenic mice. (c) Northern blot for miRNA-30c in wildtype and transgenic littermates in line B at 8 weeks of age. U6 was used as a loading control and shows similar loading. (d) Quantification of miRNA-30c overexpression by qPCR in both transgenic lines at 4 weeks of age (N≥6). (e) MiRNA-30c expression in left en right ventricular tissue of line B at 4 weeks of age (N = 6). Error bars represent s.e.m. and * denotes a p-value ≤0.05.

To examine the cell types expressing miRNA-30c in the heart *in vivo*, we performed *in situ* hybridization on hearts of wildtype mice using a Fam-labeled LNA probe against miRNA-30c (Figure 1a). This staining revealed expression of miRNA-30c in the nuclei of both cardiomyocytes (black arrowheads) and non-cardiomyocytes (grey arrowheads). Hybridization with the control probe displayed no signal. In addition, we found a diffuse pattern of miRNA-30c expression in the cytoplasm of cardiomyocytes which was not observed in sections treated with the control probe. The nuclear staining likely identifies pre-miRNA-30c while the cytoplasmic staining detects mature miRNA-30c. The expression of miRNA-30c in both cardiomyocytes and non-cardiomyocytes confirms previously reported *in vitro* studies [12], [13], which show expression in cultured neonatal myocytes and fibroblasts of the rat heart.

### MiRNA-30c transgenic mice develop severely dilated cardiomyopathy

To explore the function of miRNA-30c *in vivo*, we generated cardiomyocyte-specific miRNA-30c transgenic mice by pro-nuclear oocyte injections of a αMHC-pre-miRNA-30c expression construct (Figure 1b). Two founder lines (denoted A and B) were established and both delivered offspring in normal Mendelian ratios. Northern blot analysis of line A shows a substantial increase in pre- and mature miRNA-30c levels (Figure 1c). MiRNA-30c specific qPCR quantification revealed a ∼6- and ∼9-fold overexpression in left ventricular tissue of line A and B, respectively (Figure 1d). There was no significant difference in the level of overexpression between left and right ventricles (Figure 1e). We additionally evaluated the stability of transgenic overexpression in time and found left ventricular miRNA-30c levels to be stable both with increasing age and over at least seven generations (Figure S1a). Since high overexpression levels of miRNA-30c might interfere with the maturation process of other miRNAs, we evaluated the expression levels of a set of other cardiac miRNAs (known to be expressed by cardiomyocytes, cardiac fibroblasts or endothelial cells). We found a modest decrease in the expression of some of the miRNAs tested in the miRNA-30c transgenic mice, however this did not reach statistical significance (Figure S1b).

Having generated a stable and specific miRNA-30 overexpression model we phenotypically compared wildtype and transgenic hearts. The miRNA-30c transgenic mice of line A and B show no gross phenotypic abnormalities at 4 weeks of age, with line A lacking a clear phenotype until 20 weeks of age (Figure S2). In this regard, wildtype and transgenic littermates have similar heart weight, body weight and tibia length (Figure S2a–c). In addition, cardiac function as determined by echocardiography is preserved up till 20 weeks in line A (Figure S2d). Similarly, mice from the higher miRNA-30c expressing line B show no abnormalities at 4 weeks of age, neither in heart weight, body weight nor in cardiac function (Figure S2a–c, e).

**Figure 2:**
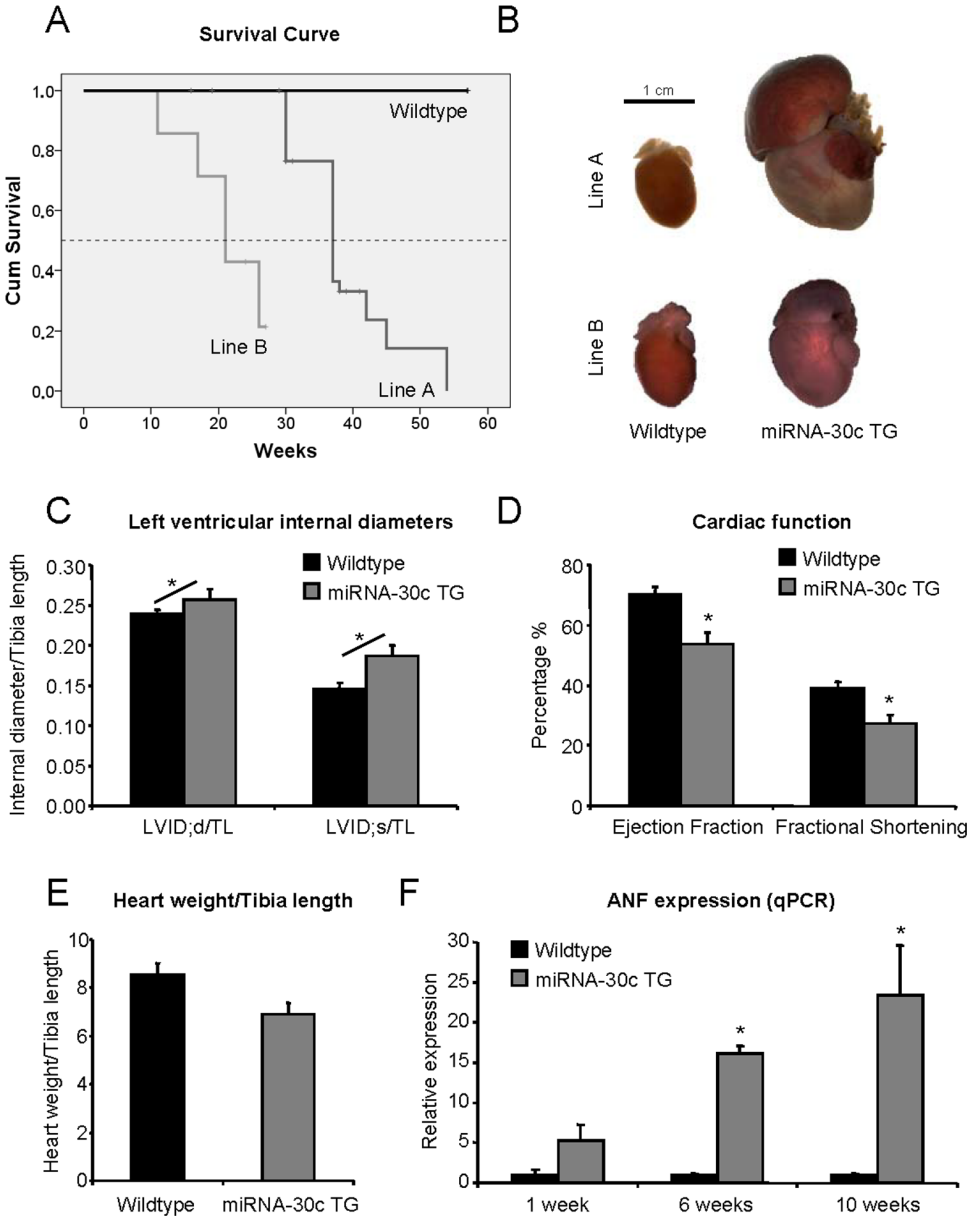
MiRNA-30c transgenic mice develop severely dilated cardiomyopathy. (a) Kaplan-Meier survival curve for both transgenic lines. We observed no mortality in wildtype littermates. 50% mortality was reached at 37 and 21 weeks for line A and B, respectively. (b) Transgenic mice of both lines develop dilated hearts with enlarged right atria during the end-stage of disease progression. In line A this occurs after an age of 12 weeks, while in line B the first signs of dilation are noted at 6 weeks of age. .(c and d) Cardiac function as determined by echocardiography in line B at 6 weeks of age (N = 3). LVID;d/TL denotes LV internal diameter during diastole, corrected for tibia length. LVID;s/TL denotes LV internal diameter during systole, corrected for tibia length. Differences between LVID;d/TL and LVID;s/TL were analysed by 2-way ANOVA for repeated measures (diagonal line indicates significant interaction effect). (e) Heart weight corrected for tibia length shows no significant difference between wildtype and miRNA-30c TG at 6 weeks of age in line B (N = 3). (f) ANF mRNA expression as evaluated by qPCR (N≥3) shows a significant increase in transgenic mice in line B. Error bars represent s.e.m. and * denotes a p-value ≤0.05.

Despite similar cardiac function at 4 weeks of age in both line A and B, miRNA-30c transgenic mice show increased mortality. As shown in Figure 2a, mortality of transgenic mice of line A and B reaches 50% at an age of 37 and 21 weeks, respectively, correlating with their relative miRNA-30c overexpression levels. Autopsy revealed cardiac dilation in combination with an enlarged right atrium (Figure 2b). Although all transgenic mice developed cardiac dilatation, the extent of the dilatation was variable from mouse to mouse in both transgenic lines. Evaluation of cardiac function by echocardiography at 6 weeks of age in line B showed that left ventricular internal diameter were increased in systole and diastole in miRNA-30c transgenic mice (Figure 2c and Figure S3). Cardiac function was decreased as evident from a reduction of the ejection fraction from 70% to 54%, and a 30% decrease in fractional shortening (Figure 2d). Surprisingly, heart weights at 6 weeks of age did not significantly differ between wildtype and miRNA-30c transgenic mice (Figure 2e, Figure S2b). Cardiac ANF expression, a marker of stress, increased with age, illustrating the progressive nature of the cardiac dysfunction (Figure 2f).

**Figure 3:**
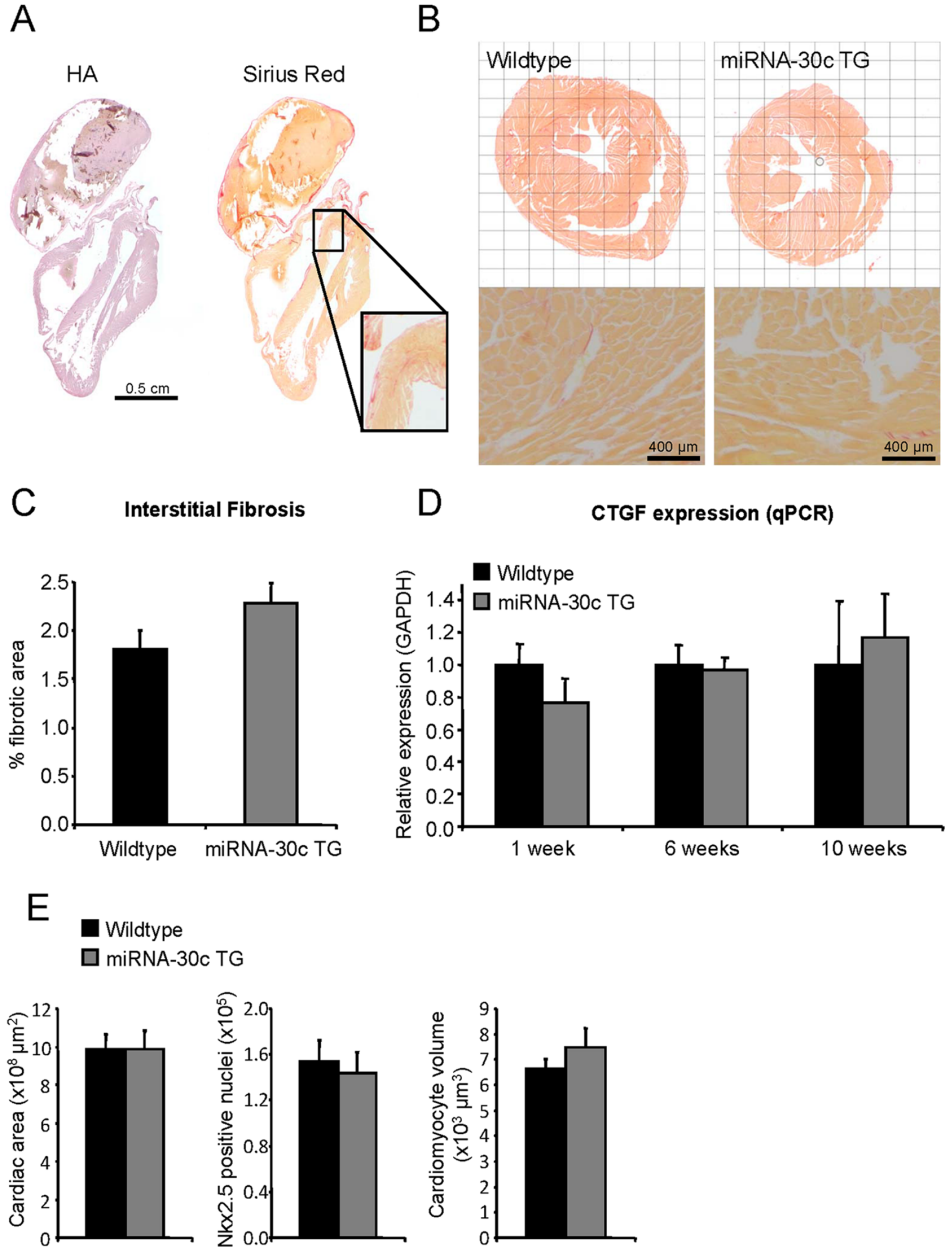
Histological analysis of miRNA-30c transgenic mice. (a) Hematoxylin-Azophloxin (HA) and sirius red staining of end-stage failing heart showing ventricular dilation and enlarged right atria in combination with interstitial fibrosis during the final stage of disease in line A. (b) Representative images of cardiac sections stained with Sirius Red show no difference in interstitial fibrosis between wildtype and transgenic mice, at 12 weeks of age in line A. (c) Quantification of interstitial fibrosis for wildtype and miRNA-30c TG mice (N≥5, 20 images per heart). (d) CTGF mRNA expression, corrected for GAPDH, at several ages in line B as evaluated by qPCR (N≥3). (e) Quantification of the cardiac area, Nkx2.5-positive nuclei and cell volumes at 4 weeks of age in line B. Quantification is based on stitched images of whole cardiac sections as shown in panel of B and Figure S4 (N≥5). Error bars represent s.e.m. and * denotes a p-value ≤0.05.

Histological analysis of cardiac tissue from failing hearts shows extensive thinning of the ventricular walls, especially in the right ventricle. The right atrium is also enlarged (Figure 3a). At this stage, stretches of interstitial fibrosis can be observed in the ventricles and the atria (Figure 3a, right panel).

### MiRNA-30c transgenic hearts show no histological abnormalities *before* onset of the phenotype

In order to investigate the underlying mechanism of the DCM phenotype we studied the hearts *before* onset of the dilated phenotype. Gross cardiac morphology was not altered in miRNA-30c transgenes (Figure 3b). In this regard, we evaluated interstitial fibrosis of the hearts before development of the dilated cardiomyopathy by quantifying Sirius red stained sections of 12 weeks old wildtype and miRNA-30c transgenic mice of line A. We could not detect significant differences in cardiac fibrosis (Figure 3b and c).

Since our laboratory previously identified CTGF as a target gene of miRNA-30c, we checked whether the 9-fold miRNA-30c overexpression in cardiomyocytes (Figure 1d) is sufficient to inhibit CTGF expression. Therefore, we quantified CTGF mRNA levels by qPCR in hearts of 1, 6 and 10 week old transgenic mice and found a small non-significant down-regulation in 1 week old hearts, but no changes at 6 or 10 weeks, when compared to wildtype littermates (Figure 3d). Since miRNA-30c was reported to induce cardiomyocyte hypertrophy *in vitro*, we quantified cardiomyocyte volume on histological sections stained with cTnI and Nkx2.5 and revealed no changes in cardiomyocyte size, an indication for the absence of cardiomyocyte hypertrophy *in vivo* (Figure 3e, Figure S4). We therefore conclude that the DCM is not secondary to the development of hypertrophy or fibrosis or cardiomyocyte hypertrophy.

### MiRNA-30c transgenic mice display mitochondrial abnormalities

In order to gain insight into the molecular mechanism of the miRNA-30c-induced dilated cardiomyopathy we performed cardiac gene expression profiling by microarrays. We analyzed 4 week old wildtype and miRNA-30c transgenic hearts from line A *before* onset of the DCM phenotype (Table S1). At 4 weeks of age we found 3010 significantly differentially expressed transcripts of which 1437 were downregulated ranging from 0.19- to 0.98-fold and 1573 were upregulated from 1.03 to 14.0-fold. *In silico* pathway analysis using DAVID functional annotation tools showed enrichment of significantly downregulated genes belonging to oxidative metabolic pathways such as the tri-carboxylic acid (TCA)-cycle and mitochondrial OXPHOS complexes (Table 1). Other downregulated pathways include genes involved in cardiac muscle contraction, dilated cardiomyopathy and MAPK signalling (Table 1). Upregulated transcripts belong to genes involved in lysosomal, tight junction and insulin signalling pathways (Table 1). Striking with regard to the observed changes in oxidative metabolic gene expression is the downregulation of 20 mitochondrial ribosomal proteins (MRPL and MRPS genes, Table S2). These transcripts encode subunits of the mitochondrial ribosome, an organelle dedicated to the translation of genes from the mitochondrial genome [32]. Moreover, we did not find several previously identified miRNA-30c targets to be regulated. In this regard, CTGF expression was decreased by 35% in the transgenic hearts at 4 weeks of age in line A but did not reach statistical significance with a p-value of 0.065. Similarly, DLL4 showed a non-significant decrease of 17% for only one out of two probes. Trp53 (p53 protein), SerpinE1 (PAI-1), MTA1, and MTTP (MTP) were not found to be differentially expressed.

**Table 1:** Differentially regulated pathways at 4 weeks of age.

Downregulated pathways (4 weeks old, p≤0,05)			
Term	Count	Genes	P-Value
Citrate cycle (TCA cycle)	7	31	2,8×10^−3^
Cardiac muscle contraction	11	78	3,3×10^−3^
Dilated cardiomyopathy	12	92	3,7×10^−3^
Acute myeloid leukemia	9	54	4,8×10^−3^
Hypertrophic cardiomyopathy (HCM)	11	84	5,7×10^−3^
MAPK signaling pathway	23	265	6,4×10^−3^
Chronic myeloid leukemia	9	76	2,6×10^−2^
Adipocytokine signaling pathway	8	67	3,7×10^−2^
Inositol phosphate metabolism	7	54	4,0×10^−2^
Oxidative phosphorylation	12	130	4,2×10^−2^
Neurotrophin signalin pathway	12	130	4,2×10^−2^
Upregulated pathways (4 weeks old, p≤0,05)			
Term	Count	Genes	P-Value
Tight junction	19	135	9,9×10^−4^
Lysosome	16	119	4,4×10^−3^
Adherens junction	12	76	5,0×10^−3^
Insulin signaling pathway	17	138	7,6×10^−3^
Wnt signaling pathway	17	149	1,5×10^−2^
Phosphatidylinositol signaling system	10	75	3,3×10^−2^
Inositol phosphate metabolism	8	54	4,0×10^−2^

Significantly down- and upregulated pathways based on the microarray expression data of left ventricular tissue in line A. Count represents the number of differentially expressed genes and Genes is the total number of genes in the given pathway.

**Figure 4:**
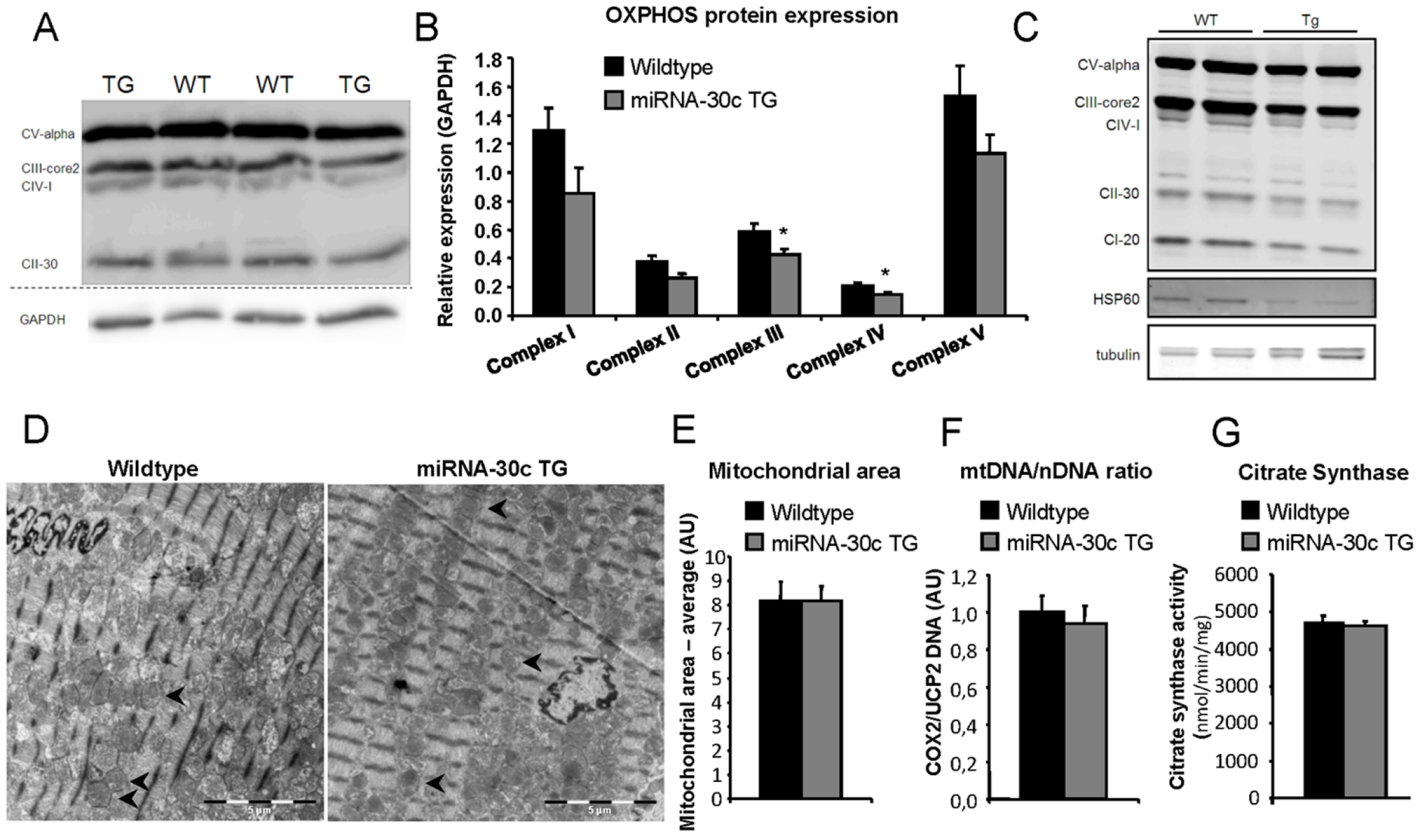
MiRNA-30c transgenic mice have a mitochondrial phenotype. (a) Western blot of mitochondrial OXPHOS proteins from wildtype and transgenic littermates, before the onset of the phenotype, at 4 weeks of age in line B. (b) Quantification of western blots of mitochondrial OXPHOS proteins of panel A (N = 6). (c) Western blot of mitochondrial OXPHOS proteins in line B at 10 weeks of age, after DCM starts to develop. (d) Overview images of electron microscopy from wildtype (left) and miRNA-30c TG mice (right) of line B. Mitochondria are indicated with a black arrowhead. (e) Quantification of mitochondrial area (N = 3, 18 images per heart) at 4 weeks of age. (f) Quantification of mitochondrial to genomic DNA ratio by qPCR (N = 4) at 4 weeks of age in line B. (g) Mitochondrial citrate synthase activity at 4 weeks of age (N = 4) in line B. Error bars represent s.e.m. and * denotes a p-value ≤0.05.

Since the microarray analysis hinted to impaired mitochondrial function we further analysed mitochondrial protein expression and morphology. We initially performed western blot assays on wildtype and transgenic hearts to quantify the protein expression levels of mitochondrial oxidative phophorylation complexes. We used the MitoProfile total OXPHOS antibody cocktail to detect complex I-20 (NDUFB8), complex II-30 (SDHB), complex III-core 2 (UQCRC3), complex IV-1 (MTCO1) and complex CV-alpha (ATP5A). This analysis revealed a ∼30% reduction in expression of the mitochondrial OXPHOS complexes before the onset of the phenotype in 4 week old mice of line B (Figure 4a–b). The loss appeared to be progressive as 10 week old transgenic mice of this line showed an even further downregulation (Figure 4c). Particularly interesting in the light of decreased mitochondrial ribosomal gene expression, and therefore impaired mitochondrial-derived mRNA translation, is the decrease of the complex IV protein MTCO1, as this protein is solely translated on the mitochondrial ribosomes. In addition, we found HSP60 protein, a mitochondrial protein chaperone, to be downregulated at 10 weeks of age in miRNA-30c transgenic mice when compared to wildtype mice (Figure 4c). In accordance with the downregulation at the protein level, the microarray analysis confirms that mRNA expression levels of HSP60 (HSPD1) and the complex I subunit (NDUFB8) are progressively downregulated, with trends for complex II (SDHB) and complex III (UQCRC2) (Table S1).

Since miRNA-30c has previously been implicated in the regulation of mitochondrial fission in neonatal cardiomyocytes *in vitro*, we performed electron microscopy on adult cardiac ventricular tissue *before* onset of the phenotype (Figure 4d). We could not observe any morphological changes indicative of impaired mitochondrial fission, as the average cross-sectional mitochondrial area was similar in wildtype and transgenes (Figure 4e).

To exclude that the changes in OXPHOS protein levels are caused by changes in the number of mitochondria, we employed two standard techniques to measure mitochondrial abundance. Quantification of the ratio between mitochondrial COX2 DNA and nuclear UCP2 DNA revealed no clear change in mitochondrial abundance between wildtype and transgenic hearts (Figure 4f). Similarly, at a functional level, activity of the TCA cycle enzyme citrate synthase was not altered in transgenic animals (Figure 4g). Both the stable DNA ratio and comparable citrate synthase activity are indicative of normal mitochondrial abundance in the transgenic hearts.

## Discussion

Since decreased cardiac miRNA-30c expression is observed in several models of overload-induced heart failure [12] but functional studies on miRNA-30c have only been done *in vitro*, we performed experiments to study its role in cardiac function *in vivo*. As the miRNA-30 family has five members, of which several have genomic duplications, a genetic knock-out approach is highly impractical. The systemic administration of antimiRs would likely result in indirect effects due to the ubiquitous expression of miRNA-30c. We therefore generated a miRNA-30c transgenic mouse model to investigate the *in vivo* effect of cardiomyocyte-specific overexpression. With this model, we show that cardiomyocyte-specific overexpression of miRNA-30c in two founder lines leads to dilated cardiomyopathy, with the onset of the cardiomyopathy correlating to the level of miRNA-30c overexpression. As mitochondrial genes were deregulated in our microarray before the onset of the phenotype we studied mitochondrial dysfunction as a possible underlying cause of the cardiomyopathy.

Mitochondria represent the power plant of a cell, since they are the source of most of the cellular ATP. They are especially abundant in metabolic active cells like myocytes. Mitochondrial oxidative respiration is a tightly regulated process that mainly takes place at the OXPHOS complexes [33]. It is not surprising that mitochondrial function is altered during acquired heart disease. During diabetic cardiomyopathy the utilization of cellular substrates shifts towards fatty acids, while glucose becomes the preferred energy substrate during the progression towards heart failure [33]. Mitochondrial morphology also changes during heart failure, as TAC-induced heart failure in rats results in decreased mitochondrial surface area [34]. Additionally, mutations in mitochondrial-encoded OXPHOS genes like Cytb, COI, COII and COIII all result in the development of a dilated cardiomyopathy [35], [36]. These observations underscore the importance of correct mitochondrial function in maintaining cardiac function.

The assembly of OXPHOS complexes requires both proper expression and cellular translocation of its constituent proteins [37], [38]. This process is complicated by the fact that some proteins derive from the nuclear genome, while others are encoded in the mitochondrial genome itself. Any imbalance in transcription, translation or mitochondrial import disturbs the normal assembly of OXPHOS complexes and interferes with mitochondrial function [39].

We show decreased mRNA expression of oxidative metabolism genes in miRNA-30c transgenic mice before the onset of the DCM phenotype. The overall ∼30% down-regulation of all five OXPHOS complexes at the protein level hints to an impaired oxidative capacity of the cardiomyocytes. The observation of decreased HSP60 protein, a chaperone in mitochondrial maintenance, and mitochondrial ribosomal subunit mRNA expression indicates potentially impaired mitochondrial homeostasis. Severe down-regulation of the mitochondrial-encoded complex IV protein MTCO1 in the later stage of DCM (10 week old miRNA-30c transgenic mice) underscores the decreased translation of mitochondrial encoded genes. Based on the ratio of mitochondrial to nuclear DNA and citrate synthase activity, we conclude, however, that mitochondrial abundance is not affected in miRNA-30c transgenics. We could not detect changes in mitochondrial area as quantified by electron microscopy. However, the net decrease of OXPHOS protein expression implies an impairment of mitochondrial function, a state that decreases the oxidative capacity of the cell and has been reported to cause DCM. It will be interesting for future studies to investigate the functional consequences of this decrease in OXPHOS protein expression on mitochondrial function and to explore whether there is an increase in oxidative stress upon miRNA-30c overexpression.

Since we observed no changes in overall mitochondrial morphology in our *in vivo* model, our results contradict the *in vitro* studies reported by Li et al. who found impaired mitochondrial fission in cultured neonatal cardiomyocytes when overexpressing miRNA-30 [17]. The proposed mechanism of mitochondrial fission *in vitro* revolves around the miRNA-30c target p53, via its effect on Drp1. However, we did not find p53 to be downregulated in our *in vivo* microarray dataset, and mitochondrial area was identical between wildtype and transgenic mice, suggesting there were no changes in mitochondrial fission.

Mitochondrial function represents only one of the cellular processes that can be regulated by miRNA-30c. Other biological processes such as fibrosis and cardiac hypertrophy could be potentially affected by miRNA-30c. In fact, miRNA-30c was initially identified as a regulator of fibrosis in cultured fibroblasts, by directly targeting CTGF [12], [40]. However, when we assessed CTGF mRNA levels at several time points in the miRNA-30c transgenic hearts, we could only find a slight but non-significant decrease at 1 week of age (Figure 3d). Quantification of interstitial fibrosis also revealed no differences before onset of the phenotype between wildtype and miRNA-30c transgenic mice. The fibrosis observed in the end-stage of the disease is likely secondary to the general cardiac dysfunction [41]. The absence of an initial effect on interstitial fibrosis, contrary to what would be expected based on *in vitro* findings from our group [12], might well result from our choice for a cardiomyocyte-specific targeting strategy. Since fibroblasts are the main effector cells of cardiac fibrosis, ECM deposition can proceed normally in cardiomyocyte-specific miRNA-30c transgenes.

In addition, previously reported *in vitro* studies with cultured neonatal rat cardiomyocytes revealed the potential of miRNA-30c to induce cardiomyocyte hypertrophy [13]. Our transgenic mice however lack a hypertrophic phenotype before onset of DCM as we found the heart weights to be stable and detected no differences in cardiomyocyte volume before onset of the phenotype. Although the hearts eventually increase in size (Figure 2a–b), this represents the DCM, likely caused by mitochondrial dysfunction. Additionally, we found no signs of altered cardiomyocyte proliferation or apoptosis as there is no difference in the density of Nkx2.5 positive nuclei between wildtype and miRNA-30c transgenic mice before onset of cardiac dilation.

The lack of a clear morphological cardiomyocyte phenotype before onset of cardiac dilation, combined with our findings on mitochondrial protein expression suggests that mitochondrial dysfunction may be the primary underlying cause of the observed dilated cardiomyopathy. One may argue that the observed phenotype might have resulted from a-specific effects due to miRNA overexpression. It is conceivable that the 6- to 9-fold overexpression of a highly expressed miRNA might deplete enzymes of the miRNA biogenesis pathway such as Drosha and Dicer. Therefore, we tested the expression of a subset of mature miRNAs but found no significant changes between wildtype and miRNA-30c transgenic mice, although there were trends to decreased expression, indicating that the overexpression of pre-miRNA-30c might slightly influence the maturation process of endogenous miRNAs. Also, the RISC complex may have become saturated with mature miRNA-30c as has been observed in the previously reported miRNA-133 transgenic mice [42], thereby altering general miRNA biogenesis. However, miRNA-133a transgenic mice show no sign of cardiac dilation while having a 13-fold induction of mature miRNA levels, with baseline expression levels as high as miRNA-30c [7]. We therefore conclude that the observed phenotype does not result from mere overexpression of any miRNA. Our findings support the hypothesis that the dilated cardiomyopathy phenotype is specifically caused by repression of miRNA-30c target genes.

Due to the multitude of predicted miRNA targets it is however unlikely that there is only one miRNA-30c target explaining the DCM phenotype. This also becomes clear from the many additional deregulated pathways we found in our gene-expression analysis. Among the miRNA-30c predicted target genes, we noted PPARγ-coactivator-α and β (PGC1 α and β), which are potentially very interesting targets since they are key regulators of mitochondrial biogenesis. Despite comprehensive experimentation, we were unable to detect any effect of miRNA-30c on mRNA and protein expression of PGC1 α and β. Also in vitro luciferase reporter assays could not reveal any direct interaction between the 3′-UTR and miRNA-30c mimics (data not shown).

In conclusion, we identified miRNA-30c as a regulator of cardiac physiology, in part via the regulation of mitochondrial function in cardiomyocytes. Our findings clearly establish the importance of proper miRNA-30c expression in maintaining normal cardiac function. With the identification of mitochondrial function as an *in vivo* process targeted by miRNA-30c we open up the path for additional studies into the role of miRNA-30c and its targets in cardiac biology and mitochondrial function.

## Supporting Information

Figure S1 **Stable expression of miRNAs in miRNA-30c transgenic mice.** (a) miRNA-30c expression as evaluated by qPCR in different generations and at different time points in miRNA-30c TG lines A and B (N≥3). Data are U6-corrected and relative to wildtype. (b) Expression of other mature miRNAs in the hearts of 10 week old mice from line B as evaluated by qPCR (N≥4). Error bars represent s.e.m. and * denotes a p-value ≤0.05.
(TIF)

Figure S2 **No phenotype at baseline in miRNA-30c transgenic mice.** (a) Heart weight (corrected for tibia length) is normal in transgenic line A at 4 and 20 weeks and in line B at 4 weeks, compared to wild-type littermates (N≥6). (b) Body weight at 4 and 20 weeks for line A and 4 weeks for line B (N≥6). (c) Tibia length at 4 and 20 weeks for line A and 4 weeks for line B (N≥6). (d) Left ventricular internal diameters during systole and diastole as quantified by echocardiography in line A at 20 weeks (N≥5). (e) Left ventricular internal diameters during systole and diastole as quantified by echocardiography in line B at 4 weeks (N = 6). Error bars represent s.e.m. and * denotes a p-value ≤0.05.
(TIF)

Figure S3 **Representative M-mode echocardiography traces of wildtype and miRNA-30c transgenic mice.** These images represent the underlying data for figure 2c-d and reveal cardiac dysfunction at 6 weeks of age.
(TIF)

Figure S4 **Overview of input for cardiac nuclear count and volume calculation for** **Figure 3E** **.** This composite image shows the three different stainings to determine the total amount of nuclei (DAPI), the cardiomyocyte nuclei (Nkx2.5), and the cardiac area (cTNI). Cardiomyocyte nuclei appear purple due to the overlay of DAPI and Nkx2.5.
(TIF)

Table S1 **Gene expression array for wildtype and transgenic mice.** Comparative analysis at 4 weeks of age (N = 3) and 20 weeks of age (N = 2) of line A. Annotated normalized data.
(XLS)

Table S2 **Extensive downregulation of subunits of the mitochondrial ribosome.** General downregulation of mitochondrial ribosomal subunits in the miRNA-30c TG mice. MRPL  =  mitochondrial ribosomal protein large; MRPS  =  mitochondrial ribosomal protein small. FC  =  Fold Change compared to wildtype littermates.
(TIF)

Table S3 **Probe and primer sequences for in situ hybridization, qPCR analysis and northern blot.**
(TIF)
