# Supplementary figures and images for: Cardiomyocyte-Specific miRNA-30c Over-Expression Causes Dilated Cardiomyopathy
Source: PLoS One. 2014 May 2;9(5):e96290. doi: 10.1371/journal.pone.0096290 (PMC4008570; doi:10.1371/journal.pone.0096290)

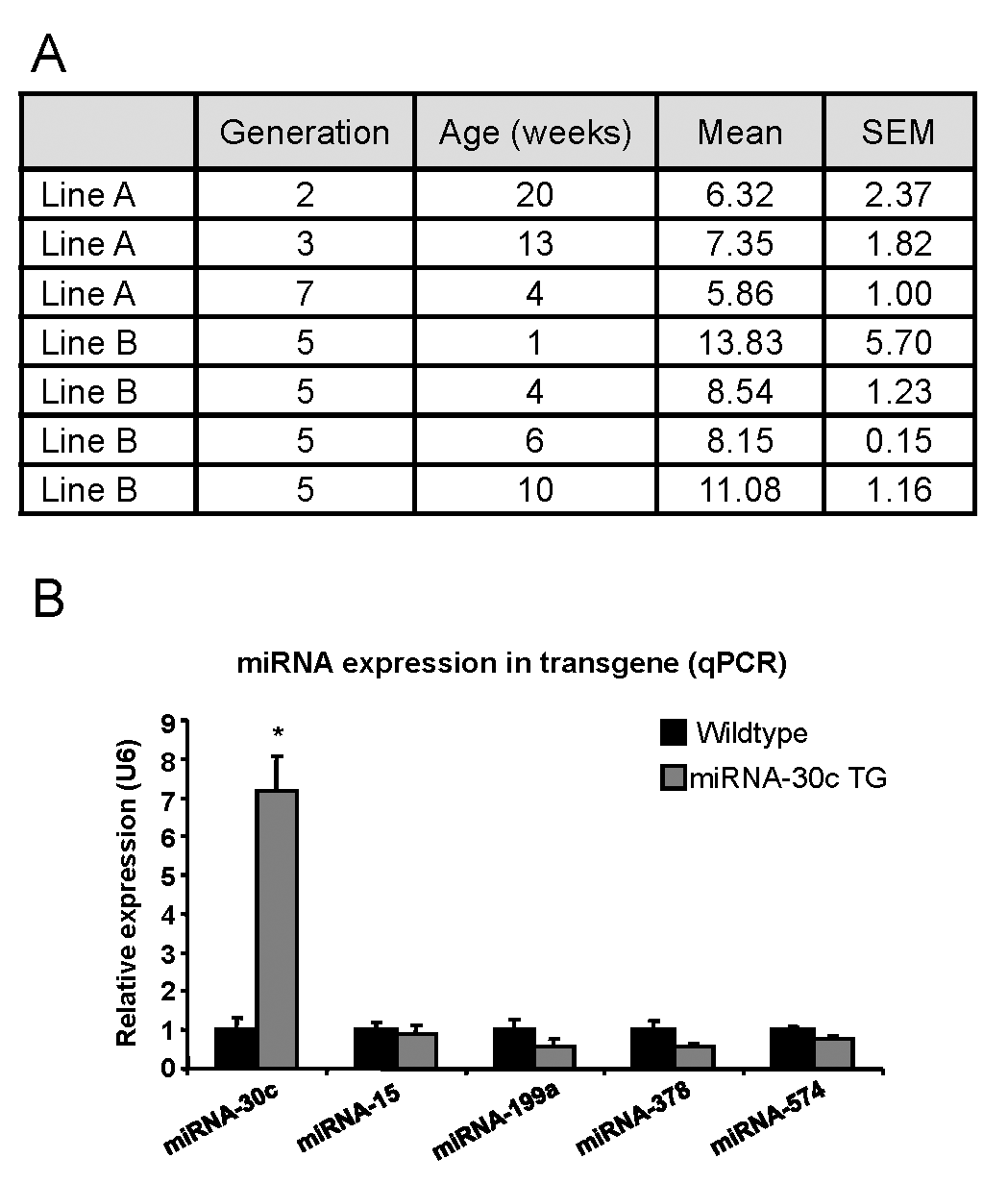

Supplement: Figure S1 — Stable expression of miRNAs in miRNA-30c transgenic mice. (a) miRNA-30c expression as evaluated by qPCR in different generations and at different time points in miRNA-30c TG lines A and B (N≥3). Data are U6-corrected and relative to wildtype. (b) Expression of other mature miRNAs in the hearts of 10 week old mice from line B as evaluated by qPCR (N≥4). Error bars represent s.e.m. and * denotes a p-value ≤0.05. (TIF) [file pone.0096290.s001.tif]

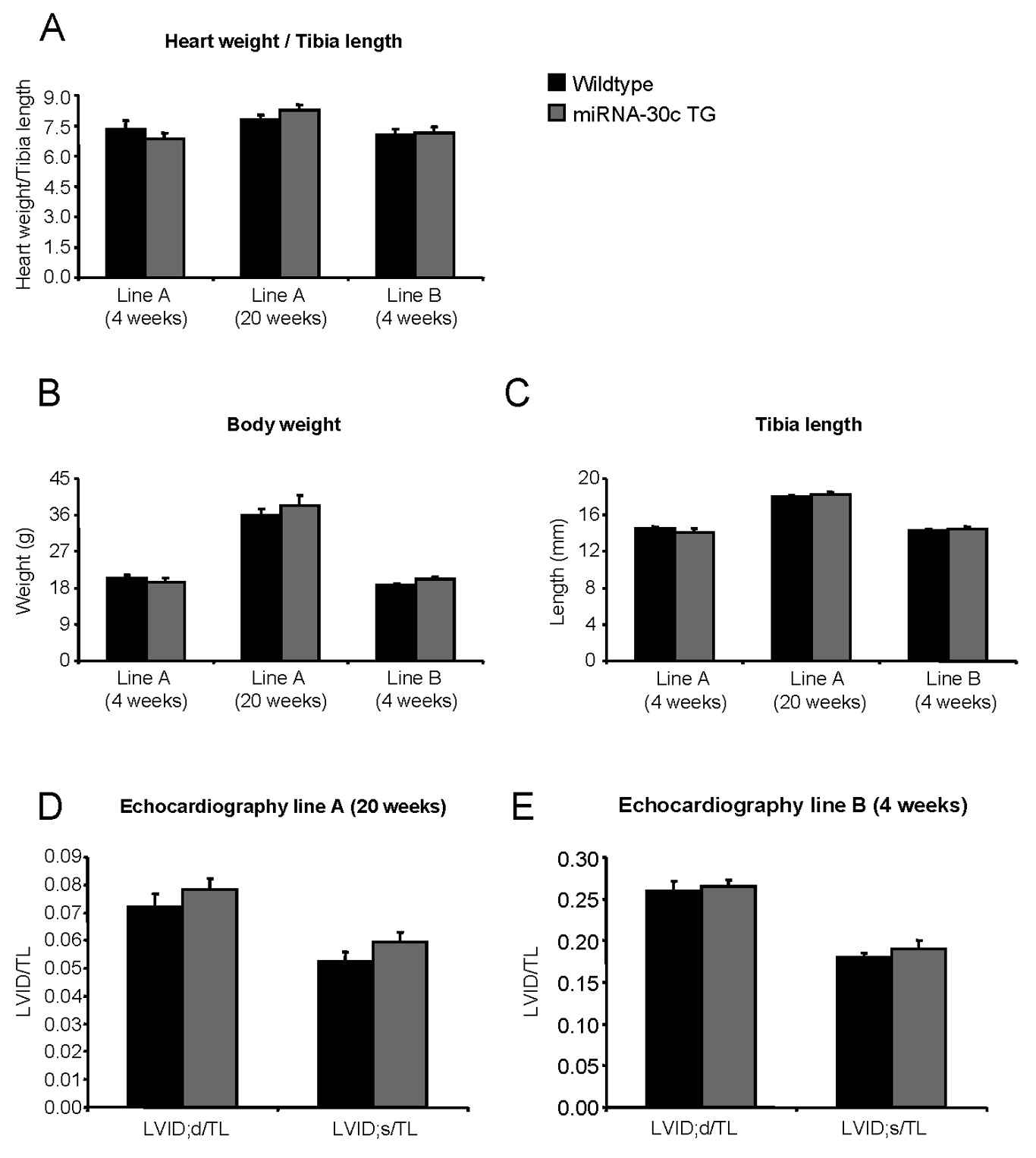

Supplement: Figure S2 — No phenotype at baseline in miRNA-30c transgenic mice. (a) Heart weight (corrected for tibia length) is normal in transgenic line A at 4 and 20 weeks and in line B at 4 weeks, compared to wild-type littermates (N≥6). (b) Body weight at 4 and 20 weeks for line A and 4 weeks for line B (N≥6). (c) Tibia length at 4 and 20 weeks for line A and 4 weeks for line B (N≥6). (d) Left ventricular internal diameters during systole and diastole as quantified by echocardiography in line A at 20 weeks (N≥5). (e) Left ventricular internal diameters during systole and diastole as quantified by echocardiography in line B at 4 weeks (N = 6). Error bars represent s.e.m. and * denotes a p-value ≤0.05. (TIF) [file pone.0096290.s002.tif]

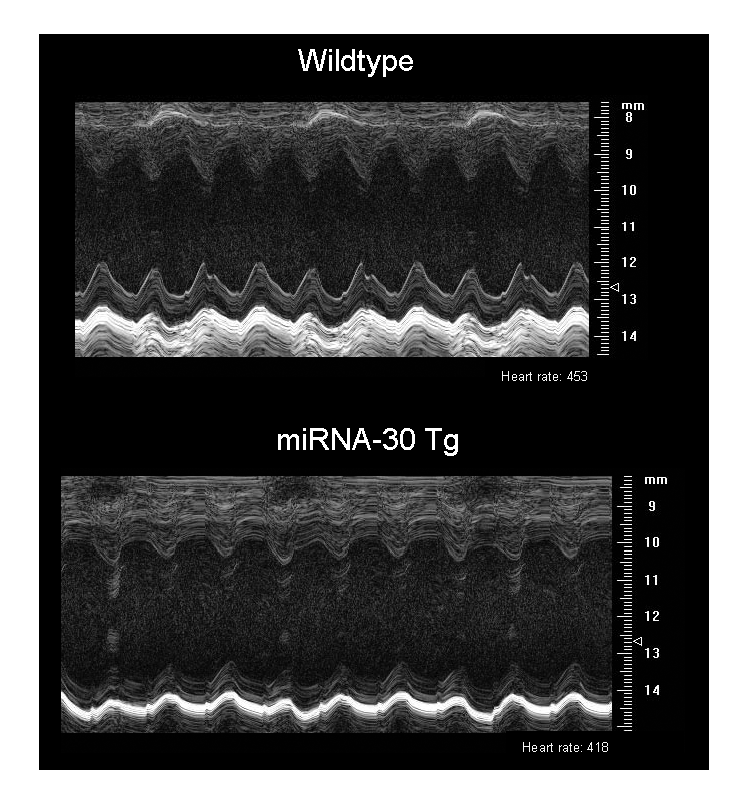

Supplement: Figure S3 — Representative M-mode echocardiography traces of wildtype and miRNA-30c transgenic mice. These images represent the underlying data for figure 2c-d and reveal cardiac dysfunction at 6 weeks of age. (TIF) [file pone.0096290.s003.tif]

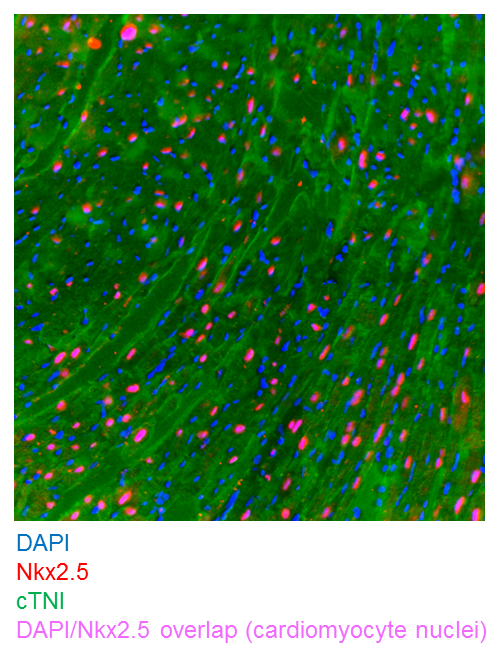

Supplement: Figure S4 — Overview of input for cardiac nuclear count and volume calculation for Figure 3E . This composite image shows the three different stainings to determine the total amount of nuclei (DAPI), the cardiomyocyte nuclei (Nkx2.5), and the cardiac area (cTNI). Cardiomyocyte nuclei appear purple due to the overlay of DAPI and Nkx2.5. (TIF) [file pone.0096290.s004.tif]

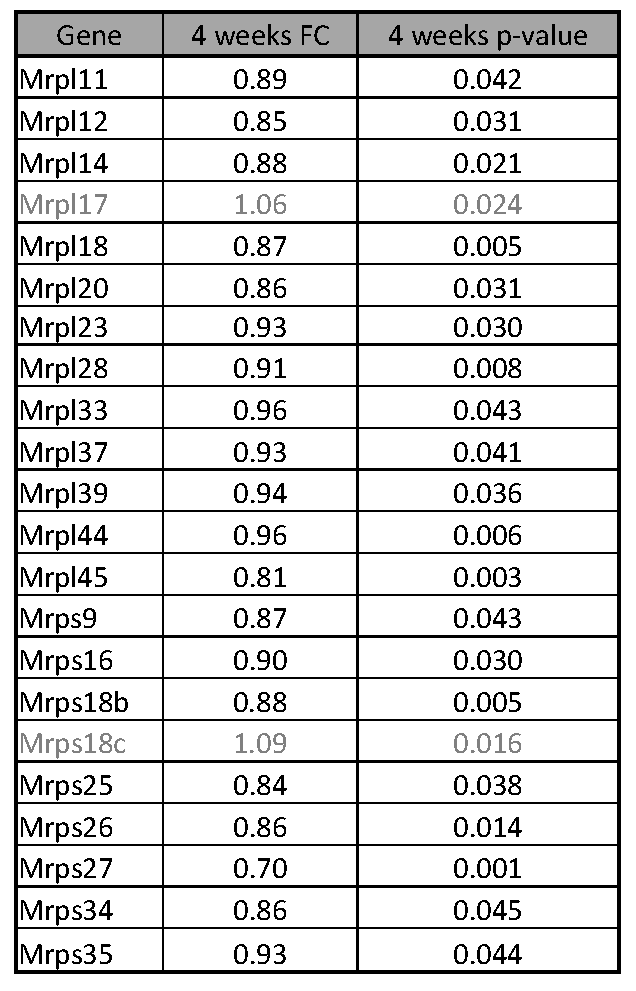

Supplement: Table S2 — Extensive downregulation of subunits of the mitochondrial ribosome. General downregulation of mitochondrial ribosomal subunits in the miRNA-30c TG mice. MRPL = mitochondrial ribosomal protein large; MRPS = mitochondrial ribosomal protein small. FC = Fold Change compared to wildtype littermates. (TIF) [file pone.0096290.s006.tif]

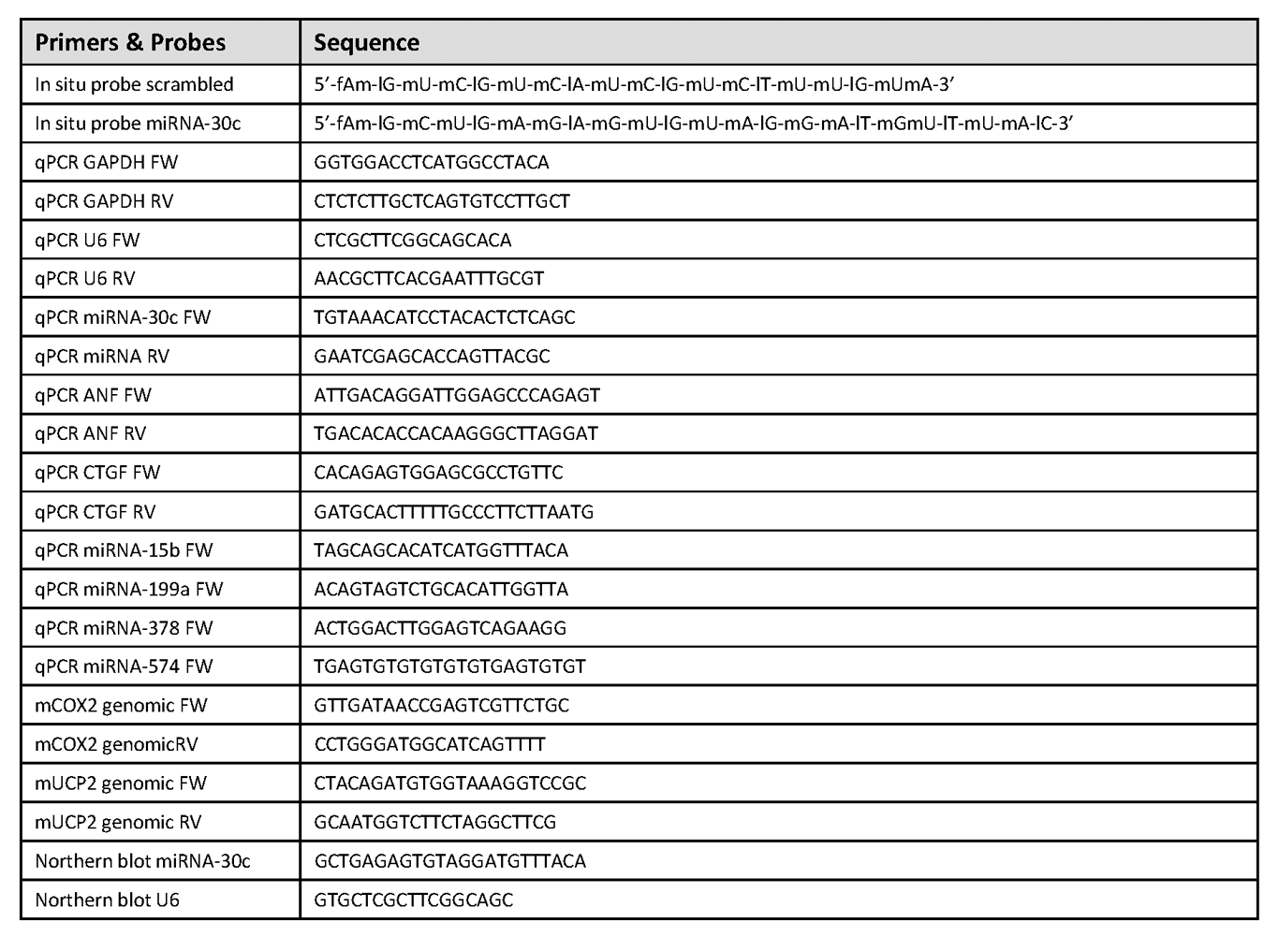

Supplement: Table S3 — Probe and primer sequences for in situ hybridization, qPCR analysis and northern blot. (TIF) [file pone.0096290.s007.tif]
